# Supplementary material for: Antibodies targeting the Crimean-Congo Hemorrhagic Fever Virus nucleoprotein protect via TRIM21
Source: Nat Commun. 2024 Oct 25;15:9236. doi: 10.1038/s41467-024-53362-7 (PMC11511847; doi:10.1038/s41467-024-53362-7)
Supplement: Supplementary file 1 — Supplementary Information [file 41467_2024_53362_MOESM1_ESM.pdf]

Supplemental Figure 1

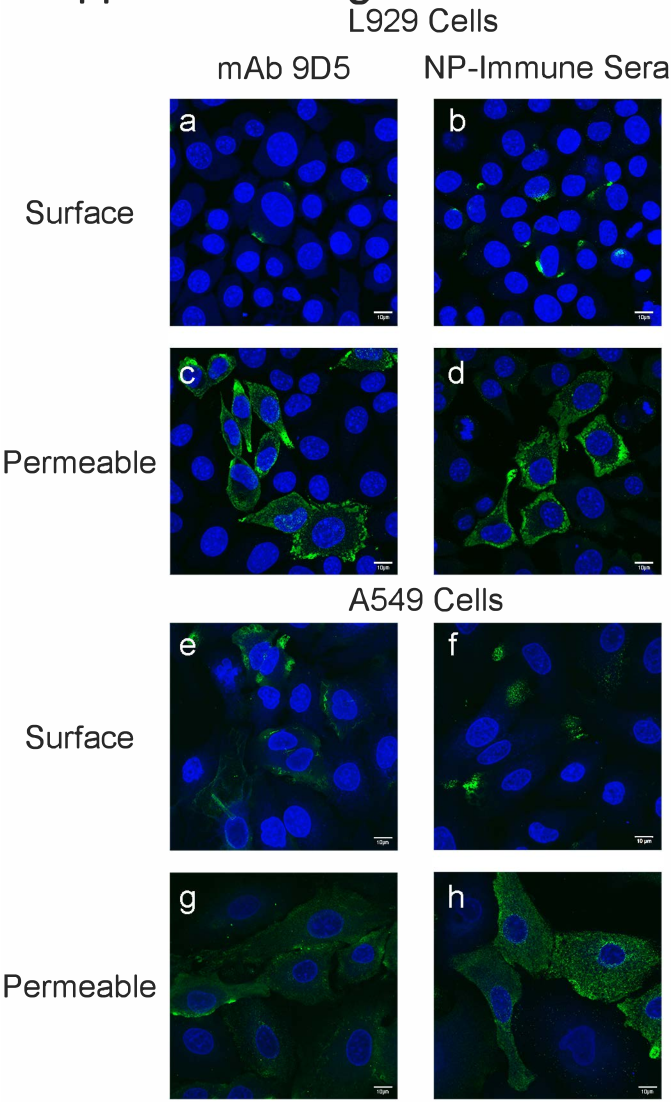

Supplemental Figure 1: Immunofluorescence of CCHFV-NP in L929 and A549 cells. L929 (a-d) and A549 (e-h) cells were infected with MA-CCHFV at an MOI 0f 1 for 24 hours. Cells were fixed with 4% paraformaldehyde and permeabilized with saponin (c-d, g-h) or unpermeabilized (a-b, e-f) and probed for CCHFV-NP using the monoclonal antibody 9D5 (left) or murine NP-immune serum as described in Figure 1 (right) and anti-mouse IgG conjugated to ALexaFluor488. Cells were also stained with NucBlue.

Supplemental Figure 2

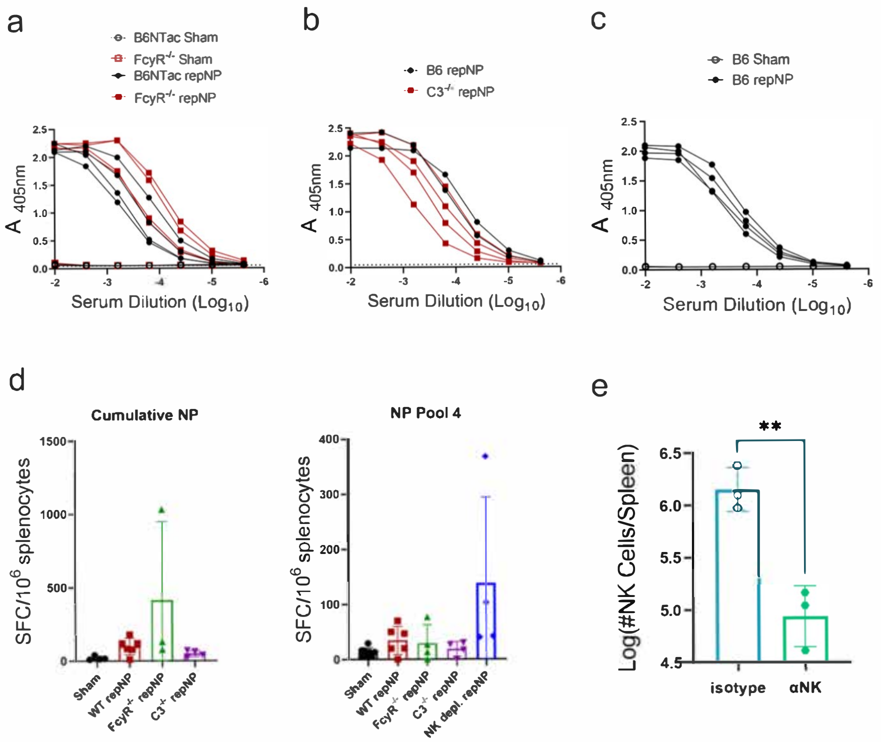

Supplemental Figure 2. repNP vaccination is immunogenic in several antibody effector function deficient mice. WT C57BL6/J or B6NTac mice, FcγR-/-, C3-/- and WT mice depleted of NK cells were vaccinated with 1ug of Sham or repNP RNA on day -28 relative to lethal CCHFV challenge. On D0, (a) FcγR-/- and control, (b) C3-/- and control, and (c) WT mice for NK depletion study were evaluated for CCHFV-specific antibody response via whole virion IgG ELISA and cellular immune response via (d) IFNy ELISpot shown as cumulative responses against peptides spanning the entire CCHFV NP or NP Pool 4. On day 9 p.i., WT mice depleted of NK cells were analyzed for (e) NK cell depletion via flow cytometry. Dashed lines indicate limit of detection. Data shown as mean plus standard deviation. Significance was calculated using one-way ANOVA; **P < 0.01.

Supplemental Figure 3

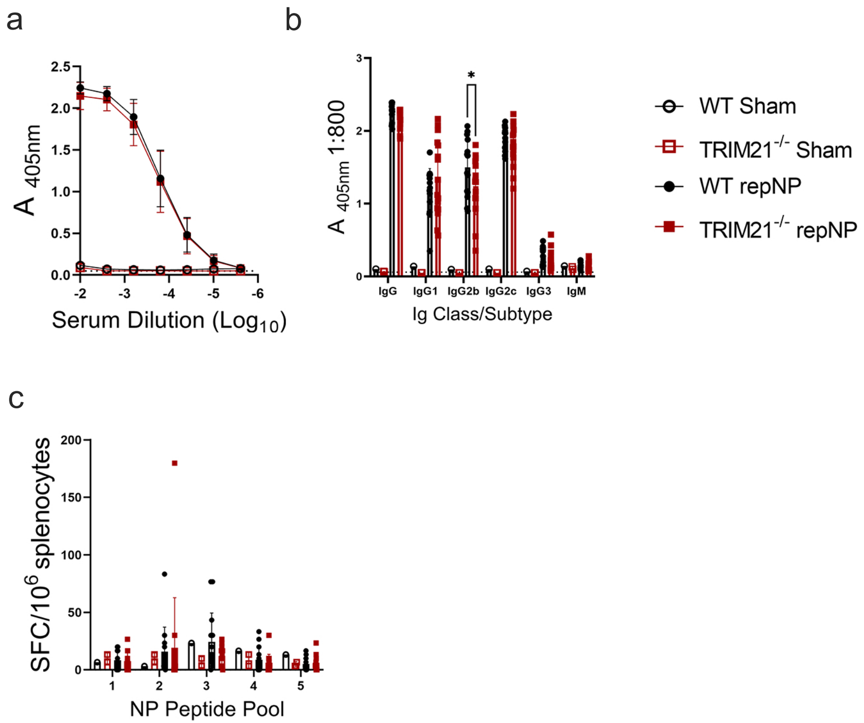

Supplemental Figure 3. repNP vaccination is immunogenic in TRIM21-/- mice. WT C57BL6/J or TRIM21-/- mice were vaccinated with 1ug of Sham or repNP RNA on day -28 relative to lethal CCHFV challenge. On D0, groups of mice were evaluated for antibody responses via (a) whole virion IgG ELISA and (b) isotype/subtype whole virion IgG ELISA. Cellular immune responses were evaluated via (c) IFNy ELISpot against CCHFV NP peptide pools (SFC: spot forming cells). Dashed lines indicate limit of detection. Significance was calculated using one-way ANOVA; *P < 0.05. Data shown as mean plus standard deviation.

Supplemental Figure 4

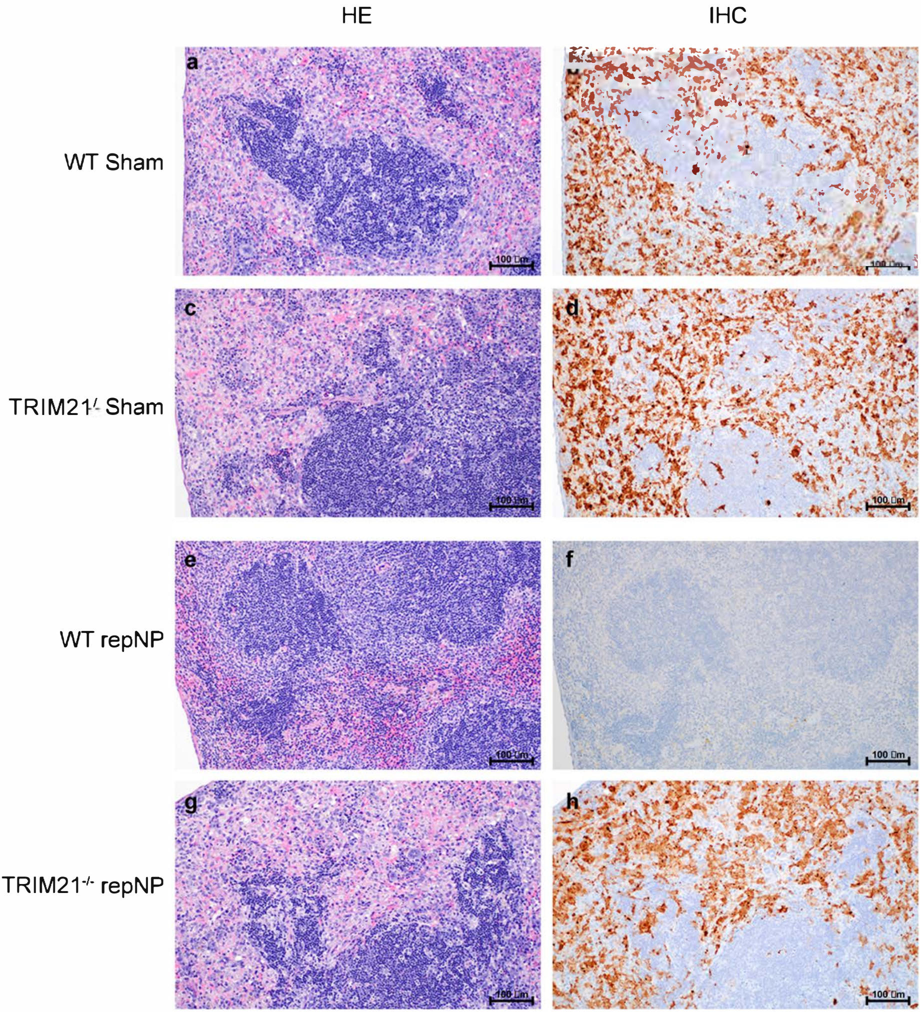

Supplemental Figure 4. repNP vaccination protects WT but not TRIM21-/- mice from spleen pathology. 200X magnification of spleen pathology from (a-b) WT sham vaccinated mice, (c-d) TRIM21-/- sham vaccinated mice, (e-f) WT repNP vaccinated mice, and (g-h) TRIM21-/- repNP vaccinated mice with (left) HE and (right) anti-CCHF IHC reactivity staining. Spleen samples from sham vaccinated and TRIM21-/- repNP vaccinated mice show reduced and apoptotic white pulp with necrotic cellular debris throughout the red pulp. Red pulp mononuclear cells (macrophages) and necrotic debris are immunoreactive. Spleen samples from WT repNP vaccinated mice are normal.

Supplemental Figure 5

a

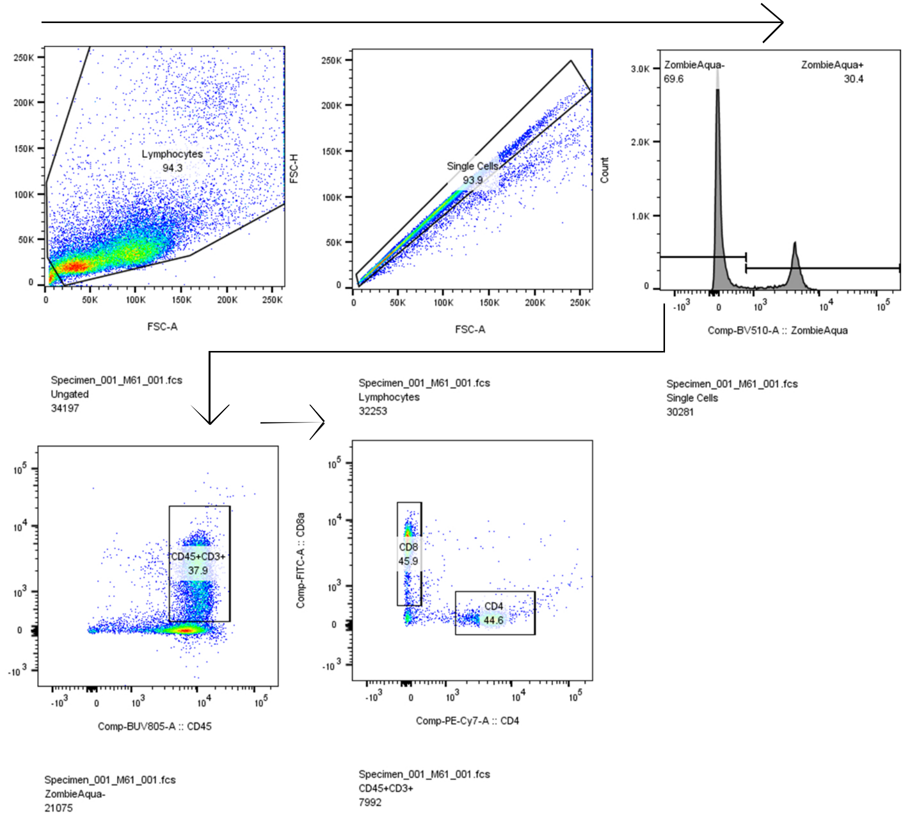

b

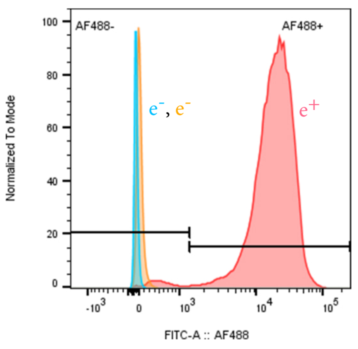

Supplemental Figure 5. Gating strategy for FLOW cytometry analyses of CD4+ and CD8+ T-cell Depletions and EDNA Assay. On D5 p.i., groups of mice (N=6) were euthanized for analyses of T-cell populations in the spleen. (a) Cells were gated on FSC and SSC to exclude debris and doublets. Dead cells were excluded via ZombieAqua viability dye. T-cells were gated by CD3+, CD45+ and CD4+ or CD8+. For the EDNA Assay, (b) L929 cells were electroporated (e+) or mixed (no electroporation, e-) with control antibody anti-mouse conjugated to AlexaFluor488 and assessed for internalization of control antibody via FLOW Cytometry gating AF488 positive and negative cells.

Supplemental Figure 6

a

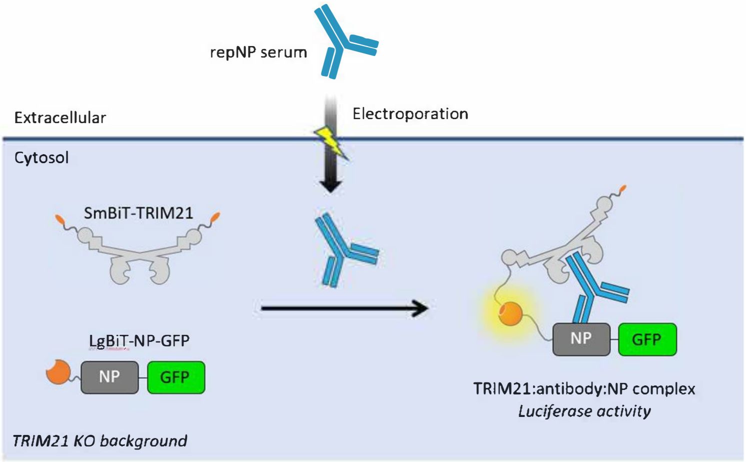

b

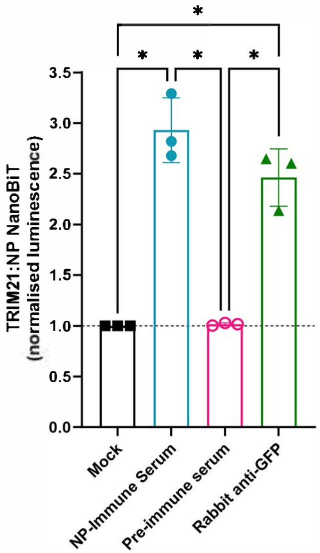

Supplemental Figure 6. NP-immune serum coordinates a complex between TRIM21 and NP in the cytosol. (a) Schematic for TRIM21:antibody:NP ternary complex assay. (B) RPE-1 TRIM21 KO cells co-expressing SmBiT-TRIM21 and LgBiT-CCHFV(Hoti)-N-mEGFP (LgBiT-NP-GFP) were electroporated with either PBS (mock), the indicated sera or anti-GFP polyclonal antibody and luminescence was measured 20 mins later. Antibodies in the NP-immune serum induce a complex between TRIM21 and NP resulting in reconstitution of luciferase activity. Graph shows mean and standard deviation (error bars) from 3 independent experiments. Statistical significance is based on one-way ANOVA; $^*P < 0.05$.

Supplemental Figure 7

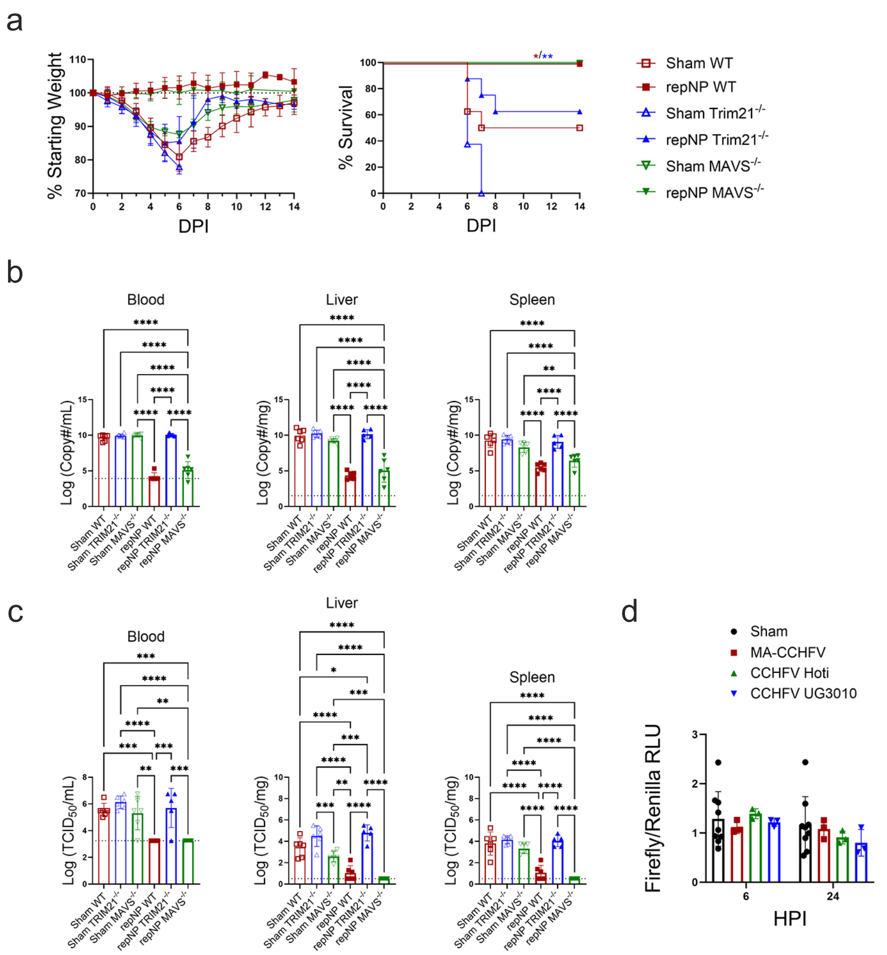

Supplemental Figure 7. repNP vaccination protects TRIM21-/- and MAVS-/- mice from lethal CCHFV infection and TRIM21-mediated protection occurs independently of the type I IFN response *in vitro.* WT C57BL6/J, TRIM21-/-, and MAVS-/- mice were vaccinated with 1ug of Sham or repNP RNA on day -28 relative to lethal CCHFV challenge. On D0, groups of mice were treated with MAR1-5A3 antibody and infected with a lethal dose of 100 TCID50 CCHFV strain UG3010. Mice (N=8) were (a) weighed daily and monitored for survival until day 14 p.i.. On D5 p.i., groups of mice (N=6) were euthanized and evaluated for (b) viral genome copies via qRT-PCR, and (c) infectious virus via TCID50 in the blood, liver, and spleen. In vitro, we assessed (d) induction of type I IFN in cells with cytoplasmic NP-specific antibody using a luciferase-reporter plasmid electroporated into cells prior to infection with MA-CCHFV, CCHFV Hoti, or CCHFV UG3010 and measurement of luciferase activity 6 and 24 hours p.i.. Plotted values are RLU ratios of Firefly/Renilla luminescence normalized to RLU ratios of mock infected samples. Dashed lines indicate limit of detection. Significance was calculated using one-way ANOVA; ns P > 0.05, *P < 0.1, **P < 0.01, ***P < 0.001, ****P < 0.0001. Data shown as mean plus standard deviation.
