# Supplementary material for: Antibodies targeting the Crimean-Congo Hemorrhagic Fever Virus nucleoprotein protect via TRIM21
Source: Nat Commun. 2024 Oct 25;15:9236. doi: 10.1038/s41467-024-53362-7 (PMC11511847; doi:10.1038/s41467-024-53362-7)
Supplement: Supplementary file 3 — Reporting Summary [file 41467_2024_53362_MOESM3_ESM.pdf]

nature portfolio

Corresponding author(s): David Hawman

Last updated by author(s): Jul 31, 2024

Reporting Summary

Nature Portfolio wishes to improve the reproducibility of the work that we publish. This form provides structure for consistency and transparency in reporting. For further information on Nature Portfolio policies, see our Editorial Policies and the Editorial Policy Checklist.

Statistics

For all statistical analyses, confirm that the following items are present in the figure legend, table legend, main text, or Methods section.

n/a	Confirmed	
☐	☒	The exact sample size (n) for each experimental group/condition, given as a discrete number and unit of measurement
☐	☒	A statement on whether measurements were taken from distinct samples or whether the same sample was measured repeatedly
☐	☒	The statistical test(s) used AND whether they are one- or two-sided<br>*Only common tests should be described solely by name; describe more complex techniques in the Methods section.*
☒	☐	A description of all covariates tested
☒	☐	A description of any assumptions or corrections, such as tests of normality and adjustment for multiple comparisons
☐	☒	A full description of the statistical parameters including central tendency (e.g. means) or other basic estimates (e.g. regression coefficient) AND variation (e.g. standard deviation) or associated estimates of uncertainty (e.g. confidence intervals)
☐	☒	For null hypothesis testing, the test statistic (e.g. F, t, r) with confidence intervals, effect sizes, degrees of freedom and P value noted<br>*Give P values as exact values whenever suitable.*
☒	☐	For Bayesian analysis, information on the choice of priors and Markov chain Monte Carlo settings
☒	☐	For hierarchical and complex designs, identification of the appropriate level for tests and full reporting of outcomes
☒	☐	Estimates of effect sizes (e.g. Cohen's d, Pearson's r), indicating how they were calculated

Our web collection on statistics for biologists contains articles on many of the points above.

Software and code

Policy information about availability of computer code

Data collection	no software was used
Data analysis	All statistics were done using GraphPad Prism.

For manuscripts utilizing custom algorithms or software that are central to the research but not yet described in published literature, software must be made available to editors and reviewers. We strongly encourage code deposition in a community repository (e.g. GitHub). See the Nature Portfolio guidelines for submitting code & software for further information.

Data

Policy information about availability of data

All manuscripts must include a data availability statement. This statement should provide the following information, where applicable:

- Accession codes, unique identifiers, or web links for publicly available datasets
- A description of any restrictions on data availability
- For clinical datasets or third party data, please ensure that the statement adheres to our policy

All data presented are available upon request.

Research involving human participants, their data, or biological material

Policy information about studies with human participants or human data. See also policy information about sex, gender (identity/presentation), and sexual orientation and race, ethnicity and racism.

Reporting on sex and gender	*Use the terms sex (biological attribute) and gender (shaped by social and cultural circumstances) carefully in order to avoid confusing both terms. Indicate if findings apply to only one sex or gender; describe whether sex and gender were considered in study design; whether sex and/or gender was determined based on self-reporting or assigned and methods used. Provide in the source data disaggregated sex and gender data, where this information has been collected, and if consent has been obtained for sharing of individual-level data; provide overall numbers in this Reporting Summary. Please state if this information has not been collected. Report sex- and gender-based analyses where performed, justify reasons for lack of sex- and gender-based analysis.*
Reporting on race, ethnicity, or other socially relevant groupings	*Please specify the socially constructed or socially relevant categorization variable(s) used in your manuscript and explain why they were used. Please note that such variables should not be used as proxies for other socially constructed/relevant variables (for example, race or ethnicity should not be used as a proxy for socioeconomic status). Provide clear definitions of the relevant terms used, how they were provided (by the participants/respondents, the researchers, or third parties), and the method(s) used to classify people into the different categories (e.g. self-report, census or administrative data, social media data, etc.) Please provide details about how you controlled for confounding variables in your analyses.*
Population characteristics	*Describe the covariate-relevant population characteristics of the human research participants (e.g. age, genotypic information, past and current diagnosis and treatment categories). If you filled out the behavioural & social sciences study design questions and have nothing to add here, write "See above."*
Recruitment	*Describe how participants were recruited. Outline any potential self-selection bias or other biases that may be present and how these are likely to impact results.*
Ethics oversight	Provided in methods

Note that full information on the approval of the study protocol must also be provided in the manuscript.

Field-specific reporting

Please select the one below that is the best fit for your research. If you are not sure, read the appropriate sections before making your selection.

☒ Life sciences ☐ Behavioural & social sciences ☐ Ecological, evolutionary & environmental sciences

For a reference copy of the document with all sections, see nature.com/documents/nr-reporting-summary-flat.pdf

Life sciences study design

All studies must disclose on these points even when the disclosure is negative.

Sample size	Animal group sizes are based on power analysis using the https://homepage.univie.ac.at/robin.ristl/samplesize.php?test=fishertest tool to see significant differences with a p value of 0.05 and power of 80%. Probabilities entered into the tool are based on previous work with these models.
Data exclusions	no data was excluded
Replication	samples were analyzed in duplicate or triplicate and data was reproduced successfully. In vivo studies were not repeated
Randomization	study animals were divided into groups randomly
Blinding	Histology was analyzed by individuals blinded towards study groups

Reporting for specific materials, systems and methods

We require information from authors about some types of materials, experimental systems and methods used in many studies. Here, indicate whether each material, system or method listed is relevant to your study. If you are not sure if a list item applies to your research, read the appropriate section before selecting a response.

Materials & experimental systems

n/a	Involved in the study	
☐	☒	Antibodies
☐	☒	Eukaryotic cell lines
☒	☐	Palaeontology and archaeology
☐	☒	Animals and other organisms
☒	☐	Clinical data
☒	☐	Dual use research of concern
☒	☐	Plants

Methods

n/a	Involved in the study	
☒	☐	ChIP-seq
☐	☒	Flow cytometry
☒	☐	MRI-based neuroimaging

Antibodies

Antibodies used	MAR1-5A3 (Leinco), anti-NK1.1 (Leinco), isotype mouse IgG2a antibody (Leinco), goat anti-mouse IgG antibody (Southern Biotech), Goat anti-mouse IgM, IgG1, IgG2b, IgG2c, and IgG3 (Southern Biotech), goat anti-mouse IgG AF488 (Thermofisher), rabbit anti-GFP polyclonal antibody (Novus; NB600-303). In house antibodies: NP-Immune sera from mice, NHP, and humans, sham sera from mice, pre-immune sera from NHP
Validation	antibodies were purchased commercially and validated by manufacturers. In-house antibodies were validated by ELISA.

Eukaryotic cell lines

Policy information about cell lines and Sex and Gender in Research

Cell line source(s)	ATCC or from collaborators Leo James and Dean Clift
Authentication	authenticated by STR profiling via ATCC (cell lines from collaborators were authenticated from ATCC test kits)
Mycoplasma contamination	cell lines were not tested for mycoplasma
Commonly misidentified lines (See ICLAC register)	n/a

Animals and other research organisms

Policy information about studies involving animals; ARRIVE guidelines recommended for reporting animal research, and Sex and Gender in Research

Laboratory animals	MAVS-/- mice on the C57BL6/J background (gift from Michael Gale were from an in-house colony. C3-/- mice on the C57BL6/J background (stock #029661), , and wild-type C57BL6/J (stock #00664) mice were purchased from Jackson Laboratories.Cryopreserved TRIM21-/- on the C57BL6/J background (stock #010724) mice were recovered at Jackson Laboratories for an in-house colony established at Rocky Mountain Laboratories, NIAID, NIH. All animals ~8weeks
Wild animals	study did not involve wild animals
Reporting on sex	both M and F animals, in equal numbers, were used
Field-collected samples	no field collected samples were used
Ethics oversight	Animal work was approved by the Rocky Mountain Laboratories Institutional Animal Care and Use Committee in accordance with recommendations by the Guide for the Care and Use of laboratory Animals of the National Institutes of Health, the Office of Animal Welfare, the United States Department of Agriculture in an association for Assessment and Accreditation of Laboratory Animal Care-Accredited Facility

Note that full information on the approval of the study protocol must also be provided in the manuscript.

Plants

Seed stocks: n/a

Novel plant genotypes: n/a

Authentication: n/a

Flow Cytometry

Plots

Confirm that:

☒ The axis labels state the marker and fluorochrome used (e.g. CD4-FITC).

☒ The axis scales are clearly visible. Include numbers along axes only for bottom left plot of group (a 'group' is an analysis of identical markers).

☒ All plots are contour plots with outliers or pseudocolor plots.

☒ A numerical value for number of cells or percentage (with statistics) is provided.

Methodology

Sample preparation: To confirm NK, CD4+ T-cell and CD8+ T-cell depletions in mice treated with depletion antibodies, spleens were harvested on day 5 p.i. during planned necropsies. For a single-cell suspension, spleens were collected into RPMI-1640 media (Thermofisher) complete with 10% FBS, benzonase, and HEPES buffered saline. Spleens were crushed against and passed through a 70-micron strainer and rinsed with additional RPMI complete. Splenocytes were pelleted and resuspended in ACK lysis buffer to lyse red blood cells. Lysis was suspended by addition of cold FACS buffer (PBS + 2% FBS) and splenocytes were pelleted, washed, and used for FLOW cytometry analyses. To assess depletion of NK cells, splenocytes were stained with ZombieAqua (Biolegend), anti-mouse NK1.1 PE (Clone PK136, Biolegend), anti-mouse CD45 BUV395 (Clone 30-F11, BD Sciences), anti-mouse CD3e BV421 (Clone 145-2C11, Biolegend), anti-mouse CD11b BV786 (Clone M1/70, Biolegend), and anti-mouse CD49b APC (Clone DX5, Biolegend). To assess depletion of CD4+ and CD8+ T-cells, splenocytes were stained with ZombieAqua (Biolegend), anti-mouse CD45 BUV805 (Clone 30-F11, BD Sciences), anti-mouse CD3 PE (Clone 145-2C11, Biolegend), anti-mouse CD4 PE/Cy7 (Clone RM4.4, Biolgened), and anti-mouse CD8a AF488 (Clone 53-6.7, BD Sciences). Cells were gated to exclude debris and non-viable cells. NK cells were defined as CD45+Cd3-CD11b+CD49b+. T-cells were defined as CD45+CD3+ and CD4+ or CD8+.

Instrument: BD FACS Symphony A5

Software: FLOWJO

Cell population abundance: See Supplemental Figure 5.

Gating strategy: See Supplemental Figure 5.

☒ Tick this box to confirm that a figure exemplifying the gating strategy is provided in the Supplementary Information.
